# Tradeoffs in viral fitness driven by alternative entry pathways

**DOI:** 10.1128/mbio.02833-25

**Published:** 2025-11-05

**Authors:** Marc Carrascosa-Sàez, Jérémy Dufloo, Rafael Sanjuán

**Affiliations:** 1 Institute for Integrative Systems Biology (I2SysBio), University of Valencia – CSIC 253325, Paterna, Spain; Charite-Universitatsmedizin Berlin ChariteCentrum 5 fur diagnostische und praventive Labormedizin, Berlin, Germany

**Keywords:** SARS-CoV-2, furin cleavage site, viral entry routes, syncytia, social evolution, virus-virus interactions

## Abstract

Enveloped viruses can enter cells either through endocytosis or by direct fusion with the plasma membrane. The latter pathway often leads to syncytia formation, caused by the fusion of infected cells with neighboring receptor-bearing cells. Yet, the implications of these two alternative entry routes for viral fitness and evolution remain poorly understood. To address this, we used a vesicular stomatitis virus-based system engineered to express the SARS-CoV-2 spike. Because deletion of the spike furin cleavage site (ΔFCS) abolishes plasma membrane fusion and restricts entry to the endocytic pathway, we compared Wuhan and ΔFCS spike variants. We found that plasma membrane entry allowed the Wuhan spike to accelerate infection and viral spread through syncytia formation. In contrast, the ΔFCS variant was less cytotoxic and reached higher titers. Coinfection assays showed that the Wuhan spike exerted a dominant negative effect on the ΔFCS mutant, outcompeting it through syncytia-mediated spread. However, when spatial segregation was enforced, the ΔFCS variant was favored instead. These findings suggest that virus-virus interactions shape the relative fitness of alternative entry routes and highlight the potential of social evolution theory to model these interactions.

**IMPORTANCE:**

Understanding how viruses enter host cells is critical for elucidating key aspects of viral infectivity, transmission, and pathogenesis. Here, we investigate the consequences of two alternative viral entry routes: endocytosis and direct entry at the plasma membrane. To this end, we employed a recombinant virus expressing the SARS-CoV-2 spike protein. This artificial system produced clear and striking phenotypes, enabling us to observe distinct differences between the two entry pathways. Plasma membrane entry promoted rapid viral spread through cell-cell fusion, whereas endocytic entry supported sustained virion production with reduced cell death. Notably, a spike variant that utilized the direct entry route dominated during coinfection, promoting extensive cell fusion and suppressing the phenotype of a variant restricted to endocytic entry. These findings clarify the functional trade-offs between viral entry pathways and introduce a novel framework for studying them through the lens of virus-virus interactions and social evolution.

## OBSERVATION

Enveloped viruses have evolved diverse strategies to enter cells, either directly at the cell surface or within endosomes. The endocytic route is followed by most viruses and is obligate for pH-dependent fusion proteins. However, some viral proteins can also trigger direct entry at the cell surface through fusion of the viral and cellular membranes (1). Moreover, such viral fusion proteins promote the formation of multinucleated cells called syncytia (2, 3). Syncytia allow cell-to-cell viral spread (4, 5), may promote antibody evasion (6), and might aid in the colonization of distant tissues (7). Syncytia have also been shown to trigger premature death (8, 9) and have been associated with increased pathogenesis and disease severity (10). However, the fitness trade-offs of these two alternative viral entry routes and syncytia formation remain poorly understood in most cases. Mutations in viral fusion proteins can impact entry route preferences, as demonstrated for instance for SARS-CoV-2 (11) and measles virus (12), suggesting that this trait can be optimized by natural selection.

Here, we use vesicular stomatitis virus (VSV) recombinant viruses expressing fluorescent reporters and the SARS-CoV-2 spike protein (S) to explore the implications of viral entry routes. This artificial system allowed us to produce extreme syncytium-inducing phenotypes and quantify differences in entry dynamics, cell death, and viral spread. We chose the SARS-CoV-2 spike because the molecular mechanisms of entry have been well elucidated. S contains a furin cleavage site (FCS) that allows activation by host furin-like enzymes and transmembrane serine protease 2 (TMPRSS2) (13), resulting in fusion of the viral and cellular membranes and viral entry directly at the plasma membrane (Fig. S1A). Alternatively, S can be activated in an FCS-independent manner by cathepsins in late endosomes (14). Hence, S can utilize both direct and endocytic entry routes in the presence of TMPRSS2 (15). In contrast, FCS ablation restricts entry to the endocytic pathway (14), thus offering a simple way to manipulate this trait (Fig. S1B).

We first checked that Wu and ΔFCS spikes were incorporated with similar efficiency into VSV particles, that spike cleavage was abolished in the ΔFCS spike, that the Wu spike mediated entry preferentially through the plasma membrane, that the ΔFCS spike-mediated entry occurred nearly exclusively through the endocytic pathway, and that the Wu spike was capable of inducing syncytia while the ΔFCS spike was not (Fig. S1C through E).

**Fig 1:**
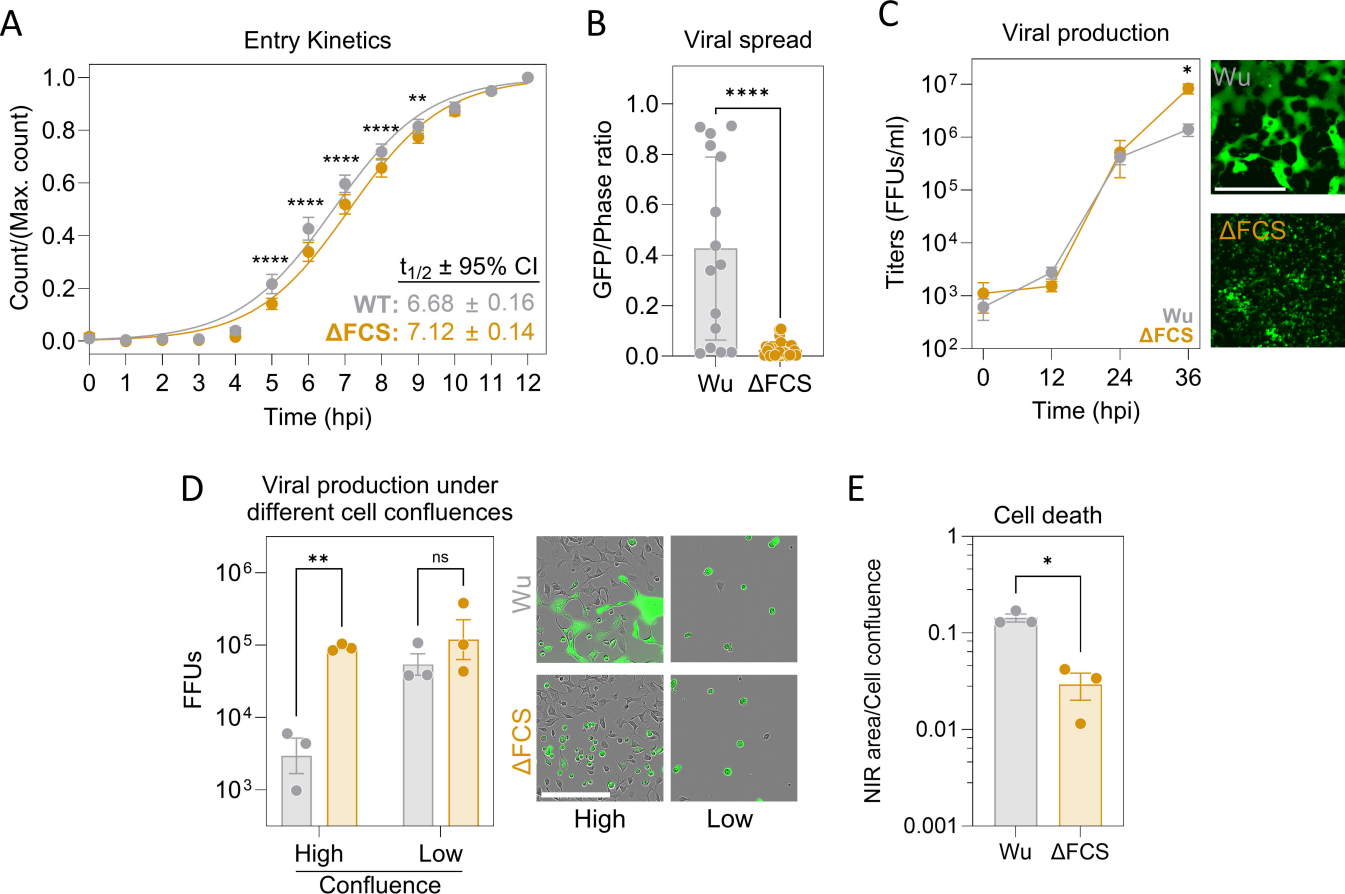
Fitness trade-offs associated with alternative entry routes and syncytia formation. (**A**) Entry kinetics. Vero E6-T cells were inoculated for 1 h at 4°C with the same amount of Wu or ΔFCS S bearing pseudotypes to allow attachment, and the temperature was shifted to 37°C to follow the GFP signal kinetics. GFP foci count was determined every hour and normalized to the maximum count. Each dot corresponds to the mean of three independent experiments (*n* = 3), each with four technical replicates. Lines correspond to a sigmoidal 2-parameter non-linear fit, which best-fit values and 95% confidence intervals for the t_1/2_ are shown. A two-way ANOVA test with repeated measures and Holm-Sidak’s multiple comparison test was performed to assess differences at each timepoint. (**B**) Viral spread. VSV recombinants capable of undergoing multiple infection cycles were obtained by cloning the Wu or ΔFCS spike genes into the VSV genome, and Vero E6-T cells were inoculated at limiting dilution in w96-plates. Viral spread was calculated as the GFP/phase ratio at 36 hpi. Sample sizes were *n* = 15 (Wu) and *n* = 26 (ΔFCS) wells with only one initially infected cell. A Mann-Whitney test for unmatched groups was performed. (**C**) Progeny yield. Vero E6-T cells were infected with each recombinant VSV at MOI 0.001 IU/cell. Supernatants were harvested and titrated every 12 h. The t_0_ corresponds to input titer. Mean + SEM from *n* = 3 independent experiments are shown, with *n* = 1 technical replicate in each independent experiment. A two-way ANOVA test with repeated measures, Geisser-Greenhouse correction for sphericity, and Holm-Sidak’s multiple comparisons test was applied. Representative images at 36 hpi are shown. Scale bar: 600 µm. (**D**) Yield of rVSV bearing either Wu or ΔFCS S in Vero E6-T cells seeded at different confluences. Cytarabine pre-treated Vero E6-T cells were infected in suspension at MOI 0.01 IU/cell and seeded in 96-well plates (80-90% confluence) or 12-well plates (5–15% confluence). Supernatants were harvested and titrated at infection plateau, and total FFUs produced were calculated. Mean + SEM from *n* = 3 independent experiments (each with 5–6 technical replicates) are shown. An ordinary two-way ANOVA test and Holm-Sidak’s test for multiple comparisons were applied. Representative images at 24 (high confluence) and 48 hpi (low confluence) are shown. Scale bar: 300 µm. (**E**) Cell death cost of syncytia formation in Vero E6-T cells. Vero E6-T cells were infected at MOI 0.001 with either Wu or ΔFCS rVSVs, and cell death was measured at 36 hpi using a NIR-Cytotox reagent. An unpaired t-test was applied. Mean from *n* = 3 independent experiments (each with 4–9 technical replicates) + SEM are shown.

We found that direct viral entry at the plasma membrane accelerated viral spread in TMPRSS2-expressing Vero E6 cells. In single-cycle experiments, the number of GFP-positive cells increased slightly faster for the Wu spike than for the ΔFCS spike (Fig. 1A), consistent with previous observations (15). Moreover, in multicycle infections, we detected a >10 fold increase in GFP signal in the Wu S-encoding virus compared to the ΔFCS variant, reflecting its ability to spread across cells more rapidly through syncytia formation (Fig. 1B).

While the Wu spike allowed faster viral spread, viral titers were lower at endpoint for VSV recombinants expressing the Wu spike than for those expressing the ΔFCS variant (Fig. 1C). This difference was probably due to syncytia formation, as both variants produced comparable progeny at low cell confluence, which prevents syncytia formation, whereas at high confluence the Wu variant yielded over 20 times less progeny (Fig. 1D). These syncytia-related fitness costs were likely due to premature cell death, as shown by cell viability assays (Fig. 1E). Nevertheless, syncytia formation may reduce viral yield through additional mechanisms. For instance, spike proteins participating in cell-cell fusion cannot be incorporated into nascent viral particles, potentially reducing their infectivity.

**Fig 2:**
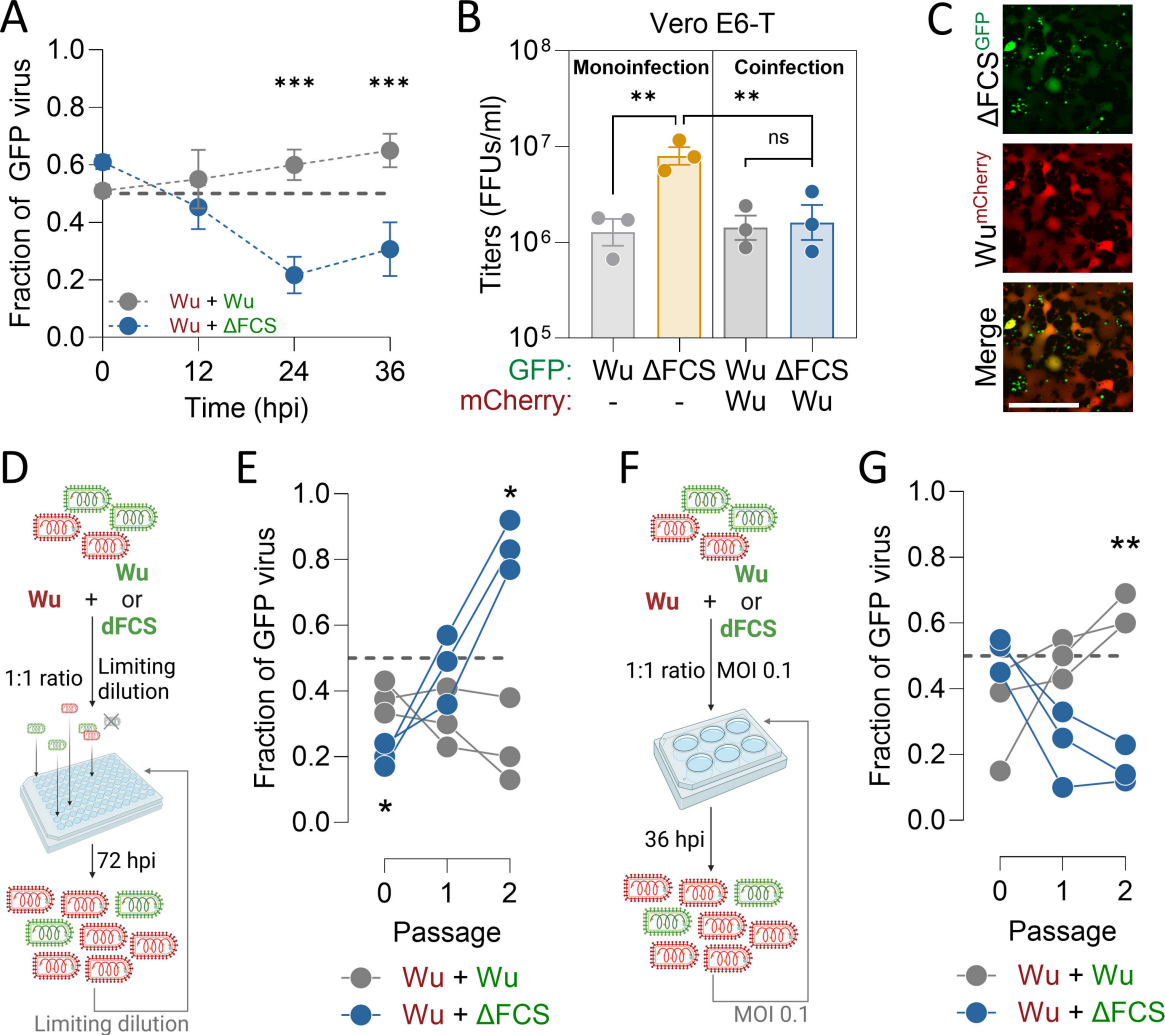
Syncytia-inducing virus exerts a negative interference over the non-syncytia inducing virus in a manner dependent on spatial structure. (**A**) VSV recombinant encoding the Cherry fluorescent marker and the Wu SARS-CoV-2 spike were engineered, and coinfections were initiated with a 1:1 ratio of the Wu (Cherry) and ΔFCS (GFP) at an MOI of 0.001 infectious units (IU) per cell. Control competition assays were carried out with Cherry and GFP viruses encoding Wu spike. Vero E6-T cells were coinfected with Wu+Wu or Wu+ΔFCS using an MOI 0.001 and initial ratio between viruses of 1:1. Supernatant was harvested and titrated every 12 h and fraction of GFP-bearing virus was calculated. Two-way ANOVA test with repeated measures and Holm-Sidak’s multiple comparisons test. Data are shown as mean ± SEM, with *n* = 3 independent experiments, with *n* = 1 technical replicate in each independent experiment. (**B**) Global titers at 36 hpi from (**A**) were compared to those in monoinfections. One-way ANOVA and Fisher’s LSD tests for multiple comparisons were performed. Data are shown as mean ± SEM, with *n* = 3 independent experiments. (**C**) Representative images of Wu+ΔFCS coinfections in Vero E6-T at 24 hpi. Scale bar: 600 µm. (**D**) Experimental setup of competition experiments in metapopulations. Spatial structure was introduced by performing infections under a limiting dilution regime in 96-well plates. Wu+ΔFCS or Wu + Wu mix was diluted so that there was 1 FFU/well on average. Supernatant was harvested 72 hpi, pooled, titrated, and diluted for a new competition passage. (**E**) The fraction of GFP-bearing virus in the pooled supernatant across two passages of competition is shown. Two-way ANOVA with repeated measures, Greisser-Greenhouse correction for unequal variances, and Holm-Sidak’s multiple comparisons test. Each series corresponds to a competition replicate, with *n* = 3 independent replicates. (**F**) Control competition experiments. Wu + ΔFCS or Wu + Wu were mixed 1:1 and used to infect Vero E6-T cells at a total MOI of 0.1. At 36 hpi, supernatant was harvested, titrated, and used to infect fresh cultures, keeping the total MOI 0.1. (**G**) The fraction of GFP-bearing rVSV for the starting virus mixture (passage 0) and two serial transfers at MOI 0.1 is shown. Two-way ANOVA with repeated measures, Greenhouse-Geisser correction for unequal variances and Holm-Sidak’s multiple comparisons test. Each series corresponds to a competition replicate, with *n* = 3 independent replicates.

In coinfections with VSV recombinants expressing the Wu (mCherry) and ΔFCS (GFP) spikes, the frequency of the ΔFCS variant decreased over time, reflecting the higher ability of the Wu variant to invade the cell population by syncytia-mediated propagation (Fig. 2A). Interestingly, viral titers at endpoint were reduced in coinfections compared to those reached by the ΔFCS alone and resembled those of the Wu variant (Fig. 2B), revealing an interference of the Wu spike over the ΔFCS variant. This interference may be due to two processes. First, the rapid spread of the Wu variant through syncytia may leave no cells available for the ΔFCS variant. Second, syncytia induction functions as a dominant trait because cells infected with the Wu spike variant can fuse with those infected with the ΔFCS variant, as evidenced by the presence of doubly fluorescent syncytia (Fig. 2C). Hence, the ΔFCS phenotype was partially suppressed in the presence of Wu spikes.

We next sought to test whether the observed interference was cell type-specific. We first used IGROV-1 cells, which express TMPRSS2 and have been used to study SARS-CoV-2 (16). The Wu variant again incurred a fitness cost in terms of titers (15.9-fold compared to the ΔFCS variant) and interfered with the ΔFCS variant in confections (Fig. S2A and C). We then used Vero E6 cells expressing little or no TMPRSS2, which should reduce syncytia and interference. As expected, syncytia were less abundant, cell death was less extensive, and the viral yields of both variants were not reduced in coinfections compared to monoinfections (Fig. S2B and C).

The above results suggest that, in our system, a spike variant that uses the endocytic route (ΔFCS) can reach higher yields, but that the direct entry route (Wu) allows faster cell-to-cell spread, is phenotypically dominant, and is favored in coinfections. In contrast, the ΔFCS may be selected if mixing was reduced. To test this, we established a metapopulation structure in which variants replicated in different compartments but were episodically mixed, simulating isolated patches within tissues, different body sites, or different hosts (Fig. 2D; Fig. S3). Competition assays showed that this setup allowed the ΔFCS variant to rapidly increase in frequency and almost displace the Wu variant (Fig. 2E through G).

Virus-virus interactions are ubiquitous in nature and have been modeled using social evolution theory (17, 18). Our results suggest that viral entry may function as a social trait because the fitness of a virus variant that uses the endocytic entry pathway is conditioned by the presence of another variant that uses the direct plasma membrane route. A similar dominant negative effect at the intercellular level has been reported in the context of innate immune evasion (19, 20). In both cases, relative fitness is modulated by interference, which in turn is dependent on spatial structure. We propose that, in future work, the evolution of alternative viral entry routes could be modeled using social evolution models such as the inclusive fitness framework and Hamilton’s rule (21).

It remains to be shown whether our findings could be validated in more relevant systems. The use of VSV, which buds at the cell surface, and a C-terminally truncated spike enhances syncytia formation compared to authentic SARS-CoV-2 (22, 23) and hence produces extreme phenotypes. Nevertheless, SARS-CoV-2 does induce syncytia *in vivo* and natural virus variants differ in their cell-cell fusion phenotypes (24). Moreover, despite the fact that deletion of the FCS is not observed in natural isolates, other mutations may have similar effects on the entry route. For instance, the Omicron variant shows lower TMPRSS2 usage and is more dependent on the endocytic entry route than the Delta variant (25). Omicron bears a H665Y substitution that is adjacent to the FCS and reduces spike fusogenicity (26), potentially contributing to the Omicron mode of entry.

Our results may apply to different types of viruses. Herpesviruses, for instance, can enter target cells directly at the cell surface or via the endocytic pathway, and trade-offs between syncytia-inducing and non-syncytia-inducing variants may exist. A single point mutation in the Cyprinid herpesvirus 3 ORF 131 increases syncytium induction and viral spread in cell cultures, and this variant interferes with and outcompetes the wild-type virus, similar to our observations (27). Similar processes may take place in Middle East Respiratory Syndrome virus (28) and measles (29). In HIV-1, envelope protein variants were initially classified according to their syncytium-inducing ability. Similar fitness trade-offs were reported in relation to this trait (30), and loss of syncytia was also found to be selectively advantageous in cell culture (31).

Overall, our results reveal new potential fitness trade-offs associated with alternative viral entry routes and suggest that virus-virus interactions may play a role in the evolution of entry routes.

## SUPPLEMENTAL MATERIAL

Supplemental Material mbio.02833-25-s0001.pdf Figures S1 to S3 and supplemental methods.

ASM does not own the copyrights to Supplemental Material that may be linked to, or accessed through, an article. The authors have granted ASM a non-exclusive, world-wide license to publish the Supplemental Material files. Please contact the corresponding author directly for reuse.
