# Supplementary material for: Tradeoffs in viral fitness driven by alternative entry pathways
Source: mBio. 2025 Nov 5;16(12):e02833-25. doi: 10.1128/mbio.02833-25 (PMC12691665; doi:10.1128/mbio.02833-25)
Supplement: Supplemental Material — Figures S1 to S3 and supplemental methods. [file mbio.02833-25-s0001.pdf]

Supplementary Materials

Supplementary Figures

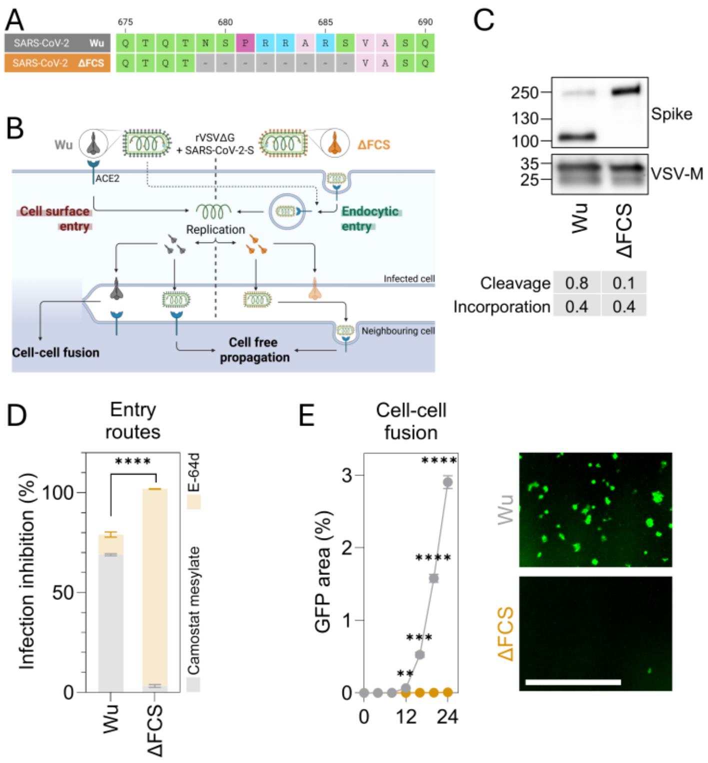

Figure S1. SARS-CoV-2 Wu and ΔFCS variants show different entry routes and syncytia formation ability.

(A) Design of the ΔFCS mutant. Comparison of the amino acid sequence around the S1/S2 cleavage site for SARS-CoV-2 Wu and ΔFCS spike variants.

(B) Schematics of each entry route and syncytia formation in the context of SARS-CoV-2 Wu and ΔFCS spikes variants.

(C) Cleavage and incorporation of spikes was assessed in viral stocks by anti-S2 Western Blot. Spike cleavage was calculated as the proportion S2/(S0+S2), and incorporation was estimated as the ratio total spike to VSV-M, (S0+S2)/M.

(D) Assessment of the preferential entry route for each variant using infection inhibition assays. Vero E6-T cells were pre-treated with 100 µM of either camostat mesylate (TMPRSS2 inhibitor) or E-64d (cathepsin L inhibitor) for 1 h and then infected with 2,000 IUs of Wu- or ΔFCS S-bearing pseudotypes. Infection inhibition (%) was calculated at 24 hpi relative to untreated controls. Camostat reduced the infectivity of Wu S-bearing VSV pseudotypes by 68.9 ± 0.9 %, whereas this reduction was only 10.1 ± 2.3 % in the presence of E-64d. In contrast, the ΔFCS pseudotypes were largely insensitive to camostat but were strongly inhibited by E-64d (98.9 ± 0.3 % infectivity reduction). Ordinary two-way ANOVA test with Holm-Sidak's multiple comparisons test. Mean + SEM for a representative experiment with n = 3 technical replicates is shown. An additional confirmatory experiment was performed with similar results (not shown).

(E) Determination of syncytia induction using a split GFP system. HEK293T-GFP1-10 and HEK293T-GFP11 were mixed 1:1 and cotransfected with ACE2 and Wu or ΔFCS spikes. GFP signal was imaged every 4 h. Representative images at 24 h post-transfection are shown. Scale

bar: 800 µm. Two-way ANOVA test with repeated measures, Geisser-Greenhouse correction for sphericity and Holm-Sidak's multiple comparisons test. Mean + SEM for a representative experiment with n = 5 technical replicates is shown. An additional confirmatory experiment was performed with similar results (not shown).

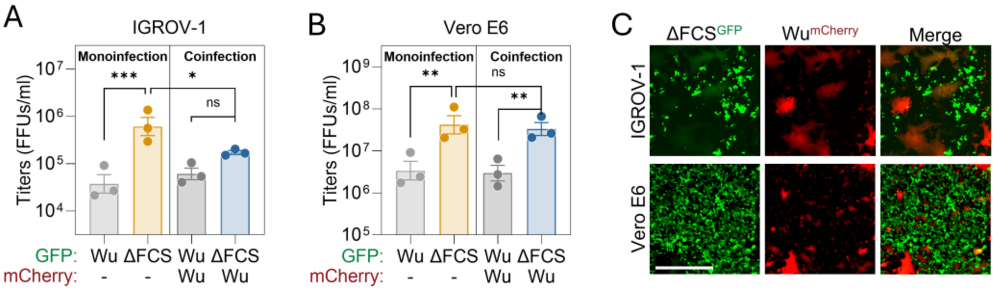

Figure S2. Interference is TMPRSS2-dependent.

(A) Monoinfections and coinfections performed in IGROV-1 cells. IGROV-1 cells were infected at an MOI of 0.001 FFUs/cell with Wu or ΔFCS variants or a 1:1 mix of Wu+Wu or Wu+ΔFCS. Supernatants were harvested 72 hpi and titrated in Vero E6-T cells. One-way ANOVA and Fisher's LSD tests for multiple comparisons were performed. Data are shown as mean ± SEM, with n = 3 independent experiments, with n=1 technical replicate in each independent experiment.

(B) Monoinfections and coinfections performed in Vero E6 cells. Cells were infected at an MOI of 0.001 FFUs/cell with Wu or ΔFCS variants or a 1:1 mix of Wu+Wu or Wu+ΔFCS. Supernatants were harvested 48 hpi and titrated in Vero E6-T cells. One-way ANOVA and Fisher's LSD tests for multiple comparisons were performed. Data are shown as mean ± SEM, with n = 3 independent experiments, with n=1 technical replicate in each independent experiment.

(C) Representative images of Wu+ΔFCS coinfections in IGROV-1 (48 hpi) and Vero E6 (48 hpi). Scale bar: 600 µm.

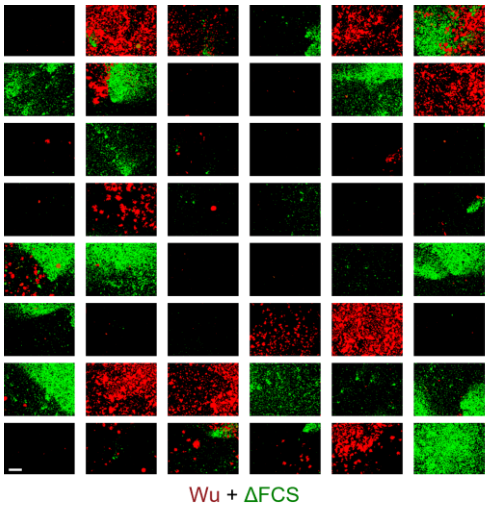

Figure S3. Competitions under limiting dilution simulate a metapopulation with reduced mixing and highly segregated viral variants. An individual replicate of passage 1 from competition experiments in metapopulations is shown at 72 hpi. GFP and mCherry signals automatically detected by the Incucyte analysis software are overlayed with green and red colors, respectively, and overlapping signals are shown in orange. Scale bar: 800 µm.

Methods

Cells

Vero E6 (a gift from Dr. Luis Enjuanes, Centro Nacional de Biotecnología), Vero E6-TMPRSS2 (JCRB Cell Bank), BHK-21 (ATCC), HEK-293T-GFP1-10 and HEK-293T-GFP-11 (a gift from Dr. Olivier Schwartz, Institut Pasteur) were grown in Dulbecco's Modified Eagle medium (DMEM, Gibco) supplemented with 10% fetal bovine serum (FBS, Gibco 10270106), 1% non-essential amino acids (Gibco), penicillin and streptomycin (10 U/ mL and 10 µg/ mL, respectively; Gibco), and amphotericin B (250 ng/ mL, Gibco). IGROV-1 cells were grown in RPMI 16640 medium (Gibco) supplemented with 10% FBS and penicillin and streptomycin (10 U/ mL and 10 µg/ mL, respectively). Additionally, 0.5mg/ml geneticin (Gibco™ 10131027) in Vero E6-T and 1µg/ml puromycin in HEK-293T-GFP1-10 and HEK-293T-GFP-11 were used. Cells were regularly tested for mycoplasma contamination by PCR.

Site directed mutagenesis

Site directed mutagenesis was performed using the NEBuilder HiFi DNA Assembly (New England Biolabs) kit and custom primers. Briefly, 1 ng of template were amplified using 250 ng of each primer pair and Phusion Plus polymerase (ThermoFisher Scientific). PCR cycling parameters were initial denaturation at 98ºC for 30 s, 35 amplification cycles of denaturation at 98ºC for 10 s, annealing at 61ºC for 10 s, and extension at 72ºC for 30 s/kb, and a final extension at 72ºC for 5 min. PCR products were then digested with DpnI (ThermoFisher Scientific) for 1 h at 37ºC, concentrated with the DNA Clean and Concentrator-5 (Zymo Research), ligated using the NEBuilder Hifi DNA Assembly kit and manufacturers' recommendations and transformed into NZY5α competent cells (Nzytech). The FCS deletion and the absence of undesired mutations in the whole plasmid were confirmed by Nanopore sequencing. Primers used were 5'-gacacagacagtggccagccagagcatc-3' and 5'-ggctggccactgtctgtgtctggtagctgg-3' for a codon-optimized version of the SARS-CoV-2 spike (used for pseudotyping), and 5'-gactcagactgtagctagtcaatccatc-3' and 5'-gactagctacagtctgagtctgataactag-3' for the non-codon-optimized version (used for constructing recombinant VSV).

VSV pseudotyping and titration

For VSV pseudotype production, approximately 8×10^6 HEK-293T cells were seeded into T75 flasks coated with poly-D-lysine (Gibco). After 16–24h, 90%-confluent monolayers were transfected with 30 µg of a His-tagged, eGFP-encoding, codon-optimized spike expression plasmid using Lipofectamine 2000 (Invitrogen) following the manufacturer's instructions. A pseudotype with no spike was produced as control by transfecting an empty pcDNA3.1 vector. At 24 h post-transfection, cells were infected with a VSVΔG+G virus at MOI = 3 IU/cell. Pseudotypes were harvested by collecting supernatants at 24 hpi, cleared by centrifugation at 2000 g for 10min, passed through a 0.45 µm filter, aliquoted and stored at −80ºC. Pseudotypes were then serially diluted in 1X DMEM supplemented with 2% FBS, preincubated 1:1 with an anti-VSV-G neutralizing monoclonal antibody (obtained in-house from a mouse hybridoma cell line) for 30 min at 37ºC and used to infect confluent cultures of Vero E6-T cells. Plates were imaged at 16-24 hpi using an Incucyte SX5 Live-Cell analysis system (Sartorius), and scored for GFP-positive cells. Titers were expressed as infectious units per mL (IU/ mL).

Western blotting

For viral particles, 1 mL of supernatant containing recombinant VSV particles was pellet by centrifugation at 30,000 g for 2 h at 4ºC and lysed in 30 µL of lysis buffer (Invitrogen, FNN0021) supplemented with cOmplete protease inhibitor (Roche) for 30 min on ice. For cell lysates, approximately 10^6 transfected cells were lysed in 80 µL of lysis buffer for 30 min on ice and then cleared by centrifugation at 15,000 g for 10 min at 4ºC. Lysates were then diluted in 4X Laemmli

buffer (Bio-Rad) supplemented with 10% β-mercaptoethanol and denatured at 95ºC for 5 min. Denatured samples were separated by sodium dodecyl sulfate-polyacrylamide gel electrophoresis in pre-cast 4-20% Mini-PROTEAN TGX gels (Bio-Rad) and then transferred onto 0.45 μm PVDF membranes (Thermo Scientific). Membranes were blocked for 1 h at room temperature (RT) in 3% bovine serum albumin TBS-T (20mM tris, 150mM NaCl, 0.1% Tween-20, pH 7.5), and then incubated for 1 h at RT with primary antibodies mouse anti-SARS-CoV S (1:100, clone 1A9, Sigma-Aldrich) and mouse anti-VSV-M (1:1,000, clone 23H12, Kerafast EB0011). Membranes were then washed three times in TBS-T, and primary antibodies were detected using goat HRP-conjugated anti-mouse IgG (1:50,000, Invitrogen, G-21040) secondary antibody. Following three washes in TBS-T, signal was revealed with SuperSignal West Pico PLUS (Thermo Scientific) and images were acquired on an ImageQuant LAS 500 (GE Healthcare) and analyzed with Fiji software (v2.1.0).

Recombinant viruses

Rescue of replication-competent recombinant VSV bearing SARS-CoV-2 Wu spike or ΔFCS mutant was performed as previously described (21, 46). Briefly, 1.5×10^5 VSV G-expressing BHK cells were seeded in 1X DMEM supplemented with 5% FBS without antibiotics in 12-well plates. The following day, 25 fmol of a plasmid containing the viral genome with the desired reporter and modifications (pVSV-reporter-ΔG-S-ΔCt, where reporter can be GFP or Cherry, S can be the Wu or ΔFCS spike, and ΔCt is a deletion of the last 21 amino acids to facilitate spike trafficking to the plasma membrane) were co-transfected along with helper plasmids encoding VSV P (25 fmol), N (75 fmol) and L (25 fmol) proteins and the T7 RNA polymerase (50 fmol) using Lipofectamine 3000 (Invitrogen) for 3 h at 37ºC. Supernatants from GFP-positive cells were harvested, cleared by centrifugation at 2000 g for 10 min, and used to infect non-VSV-G expressing Vero E6-T cells to clean viruses from VSV-G. Inoculum was removed, cells were washed 5 times with pre-warmed PBS and incubated in 1X DMEM supplemented with 2% FBS and 25% anti-VSV-G monoclonal antibody. Supernatants were harvested 48 hpi, clarified by centrifugation, aliquoted and stored at -80ºC.

Cell-cell fusion

Cell-cell fusion assay was performed as previously described (21). Briefly, 50 ng of spike expression plasmid (Wu or ΔFCS) were co-transfected with either 50 ng of human ACE2-encoding plasmid or an empty plasmid control into a 1:1 mixture of HEK-293T-GFP1-10 and HEK-293T-GFP-11 in 96-well plates (6×10^5 cells/well), using Lipofectamine 2000 (Invitrogen). Plates were incubated at 37ºC and 5% CO_2 in an Incucyte SX5 Live-Cell analysis system, and GFP and phase contrast images were taken and analyzed with the Incucyte analysis software every 4 h. The percentage of cell-cell fusion was calculated as the ratio of GFP area to cell confluence.

Selective inhibition of alternative entry routes

For inhibition of alternative entry routes, confluent Vero E6-T cells plated in 96-well plates were pre-treated with 100 μM of camostat mesylate (TMPRSS2 inhibitor, Sigma-Aldrich) or E-64d (cathepsin inhibitor, Sigma-Aldrich) for 1 h at 37ºC prior to infection. Pre-treated cells were then infected in triplicate with 2000 IUs of either pseudotype (empty control, Wu or ΔFCS), previously pre-incubated with anti-VSV-G antibody for 30 min at 37ºC. Plates were scored for GFP positive cells 24 hpi, GFP area was quantified using the Incucyte analysis software and infection inhibition was calculated as the percent relative signal reduction compared to a mock control.

Entry kinetics

Confluent Vero E6-T cells plated in 96-well plates were infected in quadruplicate with 5000 IUs either pseudotype (empty control, Wu or ΔFCS), previously pre-incubated with anti-VSV-G antibody for 30 min at 37ºC. Infection was done at 4ºC for 1 h, after which inoculum were removed

and 100 µl of 1X DMEM supplemented with 2% FBS were added to each well. Plates were placed at 37ºC and 5% CO_2 in an Incucyte SX5 Live-Cell analysis system, GFP and phase contrast images were taken every hour and analyzed using the Incucyte analysis software, and GFP positive cells (infected cells) were automatically detected. GFP count for each time was normalized to maximum GFP count, within each pseudotype. Data was fit to a sigmoidal 2-parameter non-linear function to estimate infection haltime ($t_{1/2}$) and 95% confidence interval for each entry route.

Viral spread

Vero E6-T cells were seeded in 96-well plates and infected with 1 IU/well of either Wu-S or ΔFCS-S-bearing VSV recombinant for 1 h at 37ºC. Plates were then placed at 37ºC and 5% CO2 in an Incucyte SX5 Live-Cell analysis system. GFP and phase contrast images were taken every hour, and GFP area was automatically quantified with the Incucyte analysis software. To determine spread capacity, we calculated the GFP/cell confluence ratio at 36 hpi for each positive well.

Effect of cellular confluence of viral yield

Vero E6-T cells were pre-treated overnight with 500 µM cytarabine (Supelco) to block cell division, The following day, cells were detached by trypsinization, counted and infected in suspension at an MOI of 0.01 IU/cell with either Wu or ΔFCS recombinants. Following infection, the same amount of cell:virus mix was plated either in 96-well plates or 12-well plates, achieving estimated confluences of 80-90% and 5-15%, respectively. In both cases, plates were pre-coated with poly-D-lysin and cells were seeded in 1X DMEM supplemented with 2% FBS and 500 µM cytarabine. Plates were placed at 37ºC and 5% CO_2 in an Incucyte SX5 Live-Cell analysis system, and GFP and phase contrast images were routinely acquired to follow infection progression. Supernatants were harvested and titrated at infection plateau (ca. 36 and 72 hpi, for high and low confluence conditions, respectively), and total progeny yield was calculated by correcting viral titer in the supernatant for the total volume of media (100 µL in 96-well plates or 1 mL in 12-well plates).

Growth curves

Vero E6-T cells were seeded in 6- or 12-well plates at 50% confluence. The following day, cells were inoculated at an MOI of 0.001 IU/cell at 37ºC for 1 h, after which 1X DMEM 2% FBS was added. Infection supernatant was harvested every 12 h and titrated along with input in Vero E6-T cells in 24-well plates. For titration, 100 µL of serial dilutions were used to infect confluent monolayers for 1 h at 37ºC, after which cells were overlaid with 1X DMEM 2% FBS containing 0.5% agar. Wells were then imaged every 12-24 h in an Incucyte SX5 Live-Cell analysis system, and GFP-positive foci were quantified using the Fiji software (v2.1.0).

Cell death

For analysis of cell death in infected cells, Vero E6-T cells were seeded at 50% confluence in poly-D-lysin coated 96-well plates. The following day, confluent monolayers were infected at an MOI of 0.001 either with the Wu or ΔFCS viruses, diluted in 1X DMEM containing 2% FBS and Incucyte Cytotox near infrared (NIR) Dye (1/1000 dilution; Sartorius). Infected plates were placet at 37ºC and 5% CO_2 in an Incucyte SX5 Live-Cell analysis system. GFP, NIR and phase contrast images were periodically acquired and analyzed using the Incucyte analysis software. Cell death was expressed as the ratio between NIR area and cell confluence area either at 36 or 72 hpi, for Vero E6-T and Vero E6, respectively.

Coinfections and competitions

Vero E6, Vero E6-T or IGROV-1 cells were seeded in 12-well plates, inoculated with a 1:1 mixture of each competitor, and placed at 37ºC and 5% CO_2 in an Incucyte SX5 Live-Cell analysis system. GFP, Cherry and phase contrast images were taken every 6-12 h. For each competition passage,

both input and 48, 36, and 72 hpi supernatant (for Vero E6, Vero E6-T and IGROV-1) were titrated in Vero E6-T. For competitions in Vero E6-T cells, the final titer was used to dilute the mixture and start the next competition passage keeping constant the initial MOI. For competitions at limiting dilution, Vero E6-T were seeded at 50 % confluence in 96-well plates, and infected the following day with a 1:1 mixture of the two competitors diluted such that there was only 1 IU per well on average. After infection, cultures were placed at 37ºC and 5% CO_2 in an Incucyte SX5 Live-Cell analysis system, and GFP, Cherry and phase contrast images were taken every 6-12 h. Supernatant from all wells was harvested 72 hpi, pooled, titrated, and used to start the following transfer.

Statistics

All statistical tests were performed using GraphPad Prism 10.0 software. Specific tests and samples sizes are specified in each figure legend. For all statistical tests, significance thresholds were: ns, not significant; *, p<0.05; **, p<0.01; ***, p<0.001; ****, p<0.0001.
